# Anatomic Variations of the Deep Femoral Artery and Its Branches: Clinical Implications on Anterolateral Thigh Harvesting

**DOI:** 10.7759/cureus.7867

**Published:** 2020-04-28

**Authors:** Georgia Tzouma, Nikolaos A Kopanakis, George Tsakotos, Panagiotis N Skandalakis, Dimitrios Filippou

**Affiliations:** 1 General Surgery, Metaxa Memorial Cancer Hospital, Piraeus, GRC; 2 Surgical Oncology, Metaxa Memorial Cancer Hospital, Piraeus, GRC; 3 Anatomy, National and Kapodistrian University of Athens Medical School, Athens, GRC; 4 Surgery, National and Kapodistrian University of Athens Medical School, Athens, GRC

**Keywords:** deep femoral artery, profunda femoris artery, medial circumflex femoral artery, lateral circumflex femoral artery, anterolateral thigh flap

## Abstract

The deep femoral artery (DFA) is the largest branch of the common femoral artery (CFA), supplying with its branches, the medial circumflex femoral artery (MCFA) and lateral circumflex femoral artery (LCFA), the thigh muscles, the hip joint, and the femur. Their anatomical variations have a great impact on both interventional and surgical procedures. The anterolateral thigh (ALT) flap, a versatile soft tissue with highly increasing use in reconstructive surgery, is noticeably influenced by this variability. A total of 25 articles were incorporated into the review. Studies conducted after the year 2009 were included. After the assessment of all studies included, we concluded that the DFΑ arises from the CFA with a varying site of origin, the posterolateral being the prevalent one found in 51.32% of cases. Of all cases studied, the MCFA and the LCFA most often originated from the DFA in 63.125% and 74.92%, respectively, but the CFA constitutes another frequent source of origin in 27% and 12.12% of cases, respectively. The descending branch of the lateral circumflex femoral artery (dLCFA) is the prominent pedicle in the ALT flap, originating from the LCFA in 83.55% of cases. However, the presence of an oblique lateral circumflex femoral artery (oLCFA) branch with changeable origination was observed. Knowledge of the anatomical variants in the deep femoral artery is imperative both for interventional radiologists and surgeons. Especially in reconstructive surgery, the possibility for different sources supplying the skin and the pedicle compel surgeons to acquire an awareness of this subject.

## Introduction and background

Anatomic variations of the profunda femoris or deep femoral artery (DFA) constitute a matter of great interest to anatomists, surgeons, and interventional radiologists due to their significant clinical relevance [1]. The DFA is the biggest branch of the lateral or posterior aspect of the common femoral artery (CFA) in the femoral triangle, located 2 - 6 cm below the inguinal ligament [2]. It is the main vessel for the blood supply of the adductors, flexors, and extensors thigh muscles, as well as of the hip joint and the femur [3-4]. Moreover, it plays a crucial role in the collateral blood flow between the lower pelvis and the infrapopliteal circulation [5]. The major branches of the DFA are the lateral circumflex femoral artery (LCFA) from its lateral aspect and the medial circumflex femoral artery (MCFA) from its medial wall [6]. The varying vascular anatomy of these vessels is of the utmost importance due to their involvement in vascular, orthopedic, and plastic and reconstructive surgery [7-10]. 

Knowledge of the exact origin of the LCFA is important for surgeons when applying anesthesia to the femoral nerve (FN), in orthopedic surgeries during femoral and hip procedures, when harvesting an anterolateral thigh (ALT) flap in reconstructive surgery, in aorto-popliteal bypass, in extra/intracranial bypass surgeries, but also coronary artery bypass grafting [7-9, 11-13]. Knowledge of the MCFA origin and course variations is pivotal when performing both trochanteric and intertrochanteric osteotomies, in a total arthroplasty to avoid iatrogenic avascular necrosis of the head of the femur, and during flap plastic surgery, as well as in interventional radiology during puncture of the femoral artery [14-17]. 

The ALT flap has become a particularly popular choice for reconstruction with unsurpassed utility [18-19]. The vascular supply of the ALT flap most often consists of one to three cutaneous branches of the descending branch of the lateral circumflex femoral artery (dLCFA). However, anatomical variations of the latter make the ALT flap riskier than other flaps during its harvesting, thus necessitating adequate awareness of the region [20-22]. 

This study aims to report and illustrate the variable patterns of the origin, course, and ramification of the profunda femoris artery (PFA) and its branches, as well as summarizing the literature data on this anatomical issue. 

A thorough search was conducted in the PubMed database for eligible articles from 2009 to 2020. All articles connected to DFA and ALT flap variations were entirely searched to find out all possibly pertinent information. The keywords used were the following: deep femoral artery variations, profunda femoris artery variations, MCFA variations, LCFA variations, and ALT flap variations. The search included all studies containing information about the DFA and its branches, the position of origin and morphometrics, as well as data about the vascular anatomy of the ALT flap in human subjects. The exclusion criteria were (1) articles pertaining irrelevant or inaccurate data, (2) studies containing patients with innate pathological conditions regarding the femoral region, (3) studies performed on animals, (4) articles written in languages unfamiliar to the author, and (5) articles published before the year 2009. There were no limitations imposed concerning race, age, sex, and journal. Case reports were also included. No efforts were made to search for unpublished material. 

## Review

One hundred and sixty-nine articles were initially taken into consideration. After applying the above-mentioned exclusion criteria, a sum of 25 articles was finally included in the review, analyzing a total number of 2,157 lower limbs. Those studies have covered a considerable geographical width originating from India, France, Serbia, Japan, Italy, Poland, Montenegro, Athens, Arabia, Turkey, Kenya, Africa, China, Taiwan, Singapore, Australia, Thailand, and Ohio from the United States (US). The prevalent type of study was cadaveric, while others based their results on computed tomography (CT), the computed tomography angiography (CTA) technique, and intraoperative findings during flap harvesting. The main characteristics of the studies included are depicted in Table 1.

**Table 1:** Features of Studies Included CT: computed tomography; CTA: computed tomography angiography

Study (1st author name/year/ref. #)	Country	Kind of study	Number of extremities
Manjappa et al. 2014 [5]	India	Cadaveric	40
Lalović et al. 2012 [23]	Serbia	Cadaveric	42
Zlotorowicz et al. 2018 [24]	Poland	CTA	100
Vuksanovic-Bozaric et al. 2018 [25]	Montenegro	Cadaveric	60
Sinkeet et al. 2012 [11]	Kenya	Cadaveric	84
Rajani et al. 2015 [26]	India	Cadaveric	66
Prakash et al. 2010 [27]	India	Cadaveric	64
Nasr et al. 2013 [28]	Arabia	Cadaveric	90
Rusu et al. 2017 [29]	France	CT	2
Nasu et al. 2009 [30]	Japan	Cadaveric	2
Marcucci et al. 2010 [31]	Italy	Surgical finding	1
Tsoucalas et al. 2018 [32]	Athens	Cadaveric	1
Goel et al. 2015 [33]	India	Cadaveric	1
Ciftcioğlu et al. 2009 [34]	Turkey	Cadaveric	1
Lim et al. 2017 [35]	India	CTA	513
Liu et al. 2017 [36]	China	Surgical finding	19
Lee et al. 2015 [21]	Taiwan	Surgical finding	11
Rozen et al. 2009 [37]	Australia	Surgical finding	44
Wong et al. 2009 [38]	Singapore	Surgical finding	88
Boonrod et al. 2016 [39]	Thailand	Cadaveric	50
Lu et al. 2015 [40]	Taiwan	Surgical finding	548
Seth et al. 2011 [41]	Ohio	CTA	196
Kekatpure et al. 2011 [42]	India	Surgical finding	25
Ribuffo et al. 2009 [43]	Italy	CTA	9
Wong et al. 2009 [44]	Taiwan	Surgical finding	1

Various origins of the DFA, the MCFA, and the LCFA 

**Figure 1:**
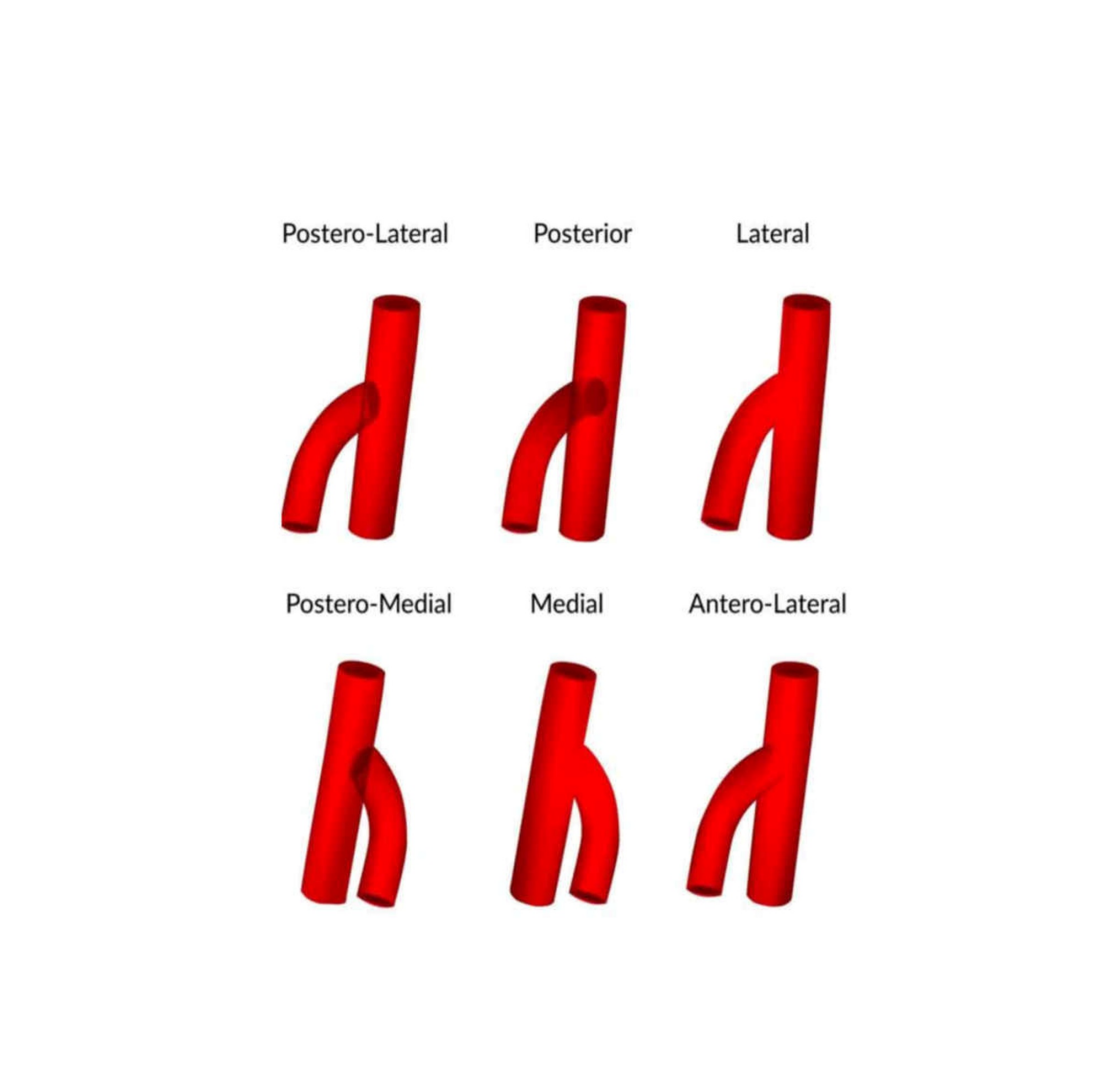
Various positions of origin of the deep femoral artery from the femoral artery Reproduced from "Variations in the origin of the deep femoral artery: A meta-analysis" by Tomaszewski et al., Clin Anat, 2017 30:106-13 [45]. Reprinted with permission.

The studies were categorized according to the site of origin of the DFA, the MCFA, and the LCFA. From the 25 studies involved, only five described the variable site of origin of the DFA from the CFA, one of them being a case report (Table 2, Figure 1) [45]. The origin of the MCFA has been the study of research in nine out of 25 studies, including three case reports, and presented high variability (Table 3) [5, 23-25, 27-30, 34]. The origin of the LCFA was illustrated in eight studies, three of which were case reports (Table 4) [5, 11, 25, 27-30, 34]. The specimens were categorized according to sex in only one study and to the side in another one. 

**Table 2:** Position of the Origin of the DFA from the CFA CFA: common femoral artery; DFA: deep femoral artery

Study	Position of origin									
	Posterolateral		Posterior		Lateral	Posteromedial		Medial	Anteromedial	
Manjappa et al. 2014 [5]	Right (R)	Left (L)	R	L	5%	5%				
	50%	70%	40%	10%		13.63%				
Rajani et al. 2015 [26]	53.03%		10.61%		18.17%			3.03%	1.51%	
Prakash et al. 2010 [27]	50%		46.9%					3.1%		
Nasr et al. 2013 [28]	Male (M)	Female (F)	20%			M	F			
	42%	42.5%				14%	10%			
Rusu et al. 2017 [29]						Right limb		Left limb		

**Table 3:** Origin of the Medial Circumflex Femoral Artery (MCFA) CFA: common femoral artery; DFA: deep femoral artery; EIA: external iliac artery; LCFA: lateral circumflex femoral artery; SFA: superficial femoral artery

Study	Origin													
	CFA			DFA				CFA as a common trunk with DFA		CFA as a common trunk with LCFA	SFA		EIA
Manjappa et al. 2014 [5]	Right (R)		Left (L)	R	L			15%					
	40%		35%	40%	60%								
Lalovic et al. 2012 [23]	33.3%			59.5%				2.4%					
Zlotorowicz et al. 2018 [24]	31%			65%							3%		
Vuksanovic-Bozaric et al. 2018 [25]	11.7%			78.3%				1.6%		1.6%	5%		
Prakash et al. 2010 [27]	32.%			67.2%									
Nasr et al. 2013 [28]	Male (M)	Female (F)		M			F	M	F		M	F	
	14%	17.5%		60%			57.5%	18%	15%		8%	10%	
Rusu et al. 2017 [29]													Right limb
Nasu et al. 2009 [30]	Left limb			Right limb									
Ciftcioglu et al. 2009 [34]	Left limb (posterolateral position)												

**Table 4:** Origin of the LCFA CFA: common femoral artery; DFA: deep femoral artery; dLCFA: descending branch of the lateral circumflex femoral artery; LCFA: lateral circumflex femoral artery; MCFA: medial circumflex femoral artery; SFA: superficial femoral artery

Study	Origin										
	CFA		DFA		CFA as a common trunk with DFA		CFA as a common trunk with MCFA	Trifurcation with DFA-MCFA	SFA		Absent
Manjappa et al. 2014 [5]	Right (R)	Left (L)	R	L	R	L					
	20%	25%	80%	70%		5%					
Vuksanovic-Bozaric et al. 2018 [25]	6.7%		83.3%		1.6%		1.6%				3.3%
Sinkeet et al. 2012 [11]	2.4%		65.55%		10.7%		14.3%	7%			
Prakash et al. 2010 [27]	18.75%		81.25%								
Nasr et al. 2013 [28]	Male (M)	Female (F)	M	F	M	F			M	F	
	8%	12.5%	74%	65%	14%	15%			4%	7.5%	
Rusu et al. 2017 [29]	Right-left side										
Nasu et al. 2009 [30]	Right side: dLCFA		Right side: remainder of LCFA; left side: LCFA								
Ciftcioglu et al. 2009 [34]			Right side								

Variability of the anterolateral thigh flap perforators 

The studies described the various origins of the main perforator of the ALT flap. From the 11 studies included, seven were clinically based on ALT flap reconstruction (one case report), three included the CTA technique, and one was cadaveric. In five studies, the dLCFA was presented as the principal vessel vascularizing the ALT flap, whereas an oblique branch was delineated in four studies. Table 5 illustrates the source vessels for the dLCFA and the oblique branch portrayed in three of the studies [35-36, 39]. 

**Table 5:** Source Vessels for the dLCFA and oLCFA CFA: common femoral artery; DFA: deep femoral artery; dLCFA: descending branch of the lateral circumflex femoral artery; oLCFA: oblique lateral circumflex femoral artery; SFA: superficial femoral artery; tLCFA: transverse branch of the lateral circumflex femoral artery

Study	Origin			
	dLCFA		oLCFA	
Lim et al. 2017 [35]	LCFA	87.1%	tLCFA	46.1%
	CFA	4.1%	dLCFA	38.2%
	DFA	5.3%	LCFA	4.5%
	SFA	3.5%	DFA	0.8%
			SFA	0.4%
Liu et al. 2017 [36]			dLCFA	87.5%
			tLCFA	12.5%
Boonrod et al. 2016 [39]	LCFA	80%		
	CFA	12%		
	DFA	8%		

A cadaveric study in the South Indian population by Manjappa et al. performed on 40 embalmed human extremities demonstrated that the DFA arose from the CFA in more than 90% of cases and from the CFA by a common trunk with the MCFA in 10% of the cases [5]. It was absent on the right side in 5% of the cases. The origin of the DFA from the mid-inguinal point (MIP) was 3.56 cm and 3.195 cm on the right and left side of the specimens, correspondingly. Concerning the position of origin, the DFA originated from the posterolateral wall of the CFA in 50% of the specimens on the right and in 70% on the left limb. The second most frequent position was the posterior (40% on right, 10% on left), followed by the lateral (5%), and the posteromedial position (5%). The MCFA arose from the CFA in 50% of the right limbs and 35% of the left limbs, in a mean distance from the MIP of 2.71 cm and 2.65 cm, respectively. The origin of the MCFA was from the DFA in 40% of the right and 60% of the left specimens, being at a distance of 3.96 and 4.85 cm from the MIP, respectively. It arose from the CFA as a common trunk with the DFA in 15% of cases. The most common site of origin was the medial site, whereas the posterior site was the least common. As for the LCFA, its source of origin appeared to be the DFA in 80% of the right specimens at a distance of 5.63 cm from the MIP and in 70% of the left specimens at a distance of 5.37 cm. It was from the CFA in a lower percentage, at a distance of 5.63 cm and 5.37 cm from the MIP on the right and left sites, respectively. The most frequent site of origin for the LCFA was the lateral site in 75% of the right and 100% of the left limbs. 

Another cadaveric study conducted on 42 thighs in Serbia by Lalovic et al. illustrated that the MCFA originated from the DFA in 59.5% of cases, at a mean distance of 57.9 ± 19.5 mm from the MIP [23]. It arose from the FA in 33.3% of cases, being at a distance of 44.2 ± 13.5 mm from the MIP. The least usual type of origin was by a common trunk with the DFA (2.4%). It was absent in 4.8% of specimens. There was no statistically significant difference between the right and left limbs or males and females. 

A study carried out by Zlotorowicz et al. in Poland analyzed 100 CTAs and concluded that both the MCFA and the LCFA derived from the DFA in 50% of the extremities [24]. In 31% of the cases, the MCFA arose from the CFA or the superficial femoral artery (SFA), whereas the LCFA arose from the DFA. In another 15% of the extremities, the LCFA originated from the CFA or the SFA, while the MCFA originated from the DFA. In two cases, they both arose from the CFA, whereas in one case, they both derived from the CFA by a common trunk. In one case, the MCFA was completely absent. 

The microdissection performed on 30 fetuses in Montenegro by Vuksanovic-Bozaric et al. depicted that the most frequent origin of the MCFA was from the DFA (78.3%), followed by the CFA (11.7%), and the SFA (5%) [25]. As for the LCFA, in 83.3% of the cases, it arose from the DFA, in 6.7% from the CFA, and in 3.3% of cases, it was not apparent at all. Noticeably, in 1.7% of the cases, the DFA arose from the external iliac artery (EIA) with the LCFA originating from the DFA and the MCFA from the SFA. 

The cadaveric study conducted in a Kenyan population on 42 cadavers by Sinkeet et al. illustrated that the LCFA was the first branch of the DFA in 65.55% of the cases, whereas in 34.5%, the origin was variant: from a common trunk with the MCFA, a common trunk with the DFA, a trifurcation with the DFA and the MCFA or from the FA [11]. 

The cadaveric study performed on 33 adult cadavers by Rajani et al. in India indicated that the most frequent amplitude of origin of the DFA from the MIP was from 21 to 40 mm on the right limb and 11 to 40 mm on the left limb. It also demonstrated that the site of origin of the DFA was mostly posterolateral (53.03%) and lateral (18.17%), with the least observed position being the posterior [26]. 

Another Indian cadaveric study on 32 properly prepared cadavers by Prakash et al. showed that the DFA originated in the proximal third of the femoral triangle in 45.3% of cases, in the middle third in 39.4%, and in the distal third in 15.1% [27]. The most frequent position of the origin of the DFA was posterolateral (50%), followed by the posterior (46.9%). Regarding the MCFA, it arose from the DFA in 67.2% of limbs and from the FA in 32.8%. As for the LCFA, its origin was from the DFA in 81.25% and the FA in 18.75%. 

The Arabian study by Nasr et al. included 45 adult human cadavers and revealed that the DFA arose from the FA with the most common site of origin being the posterolateral one, followed by the posterior side [28]. As for the pattern of origin of the MCFA, this originated from the DFA in 60% of males and 57.5% of females, with less encountered types from the FA by a common trunk with the DFA, from the FA superior to the DFA and the SFA. In respect of the LCFA, this arose mostly from the DFA, whereas less frequent types were from the FA by a common stem with the DFA, from the FA above the DFA and the SFA. The range of the main distance of the DFA from the MIP was 25 - 67 mm in males and 28 - 70 in females, whereas the distance of the MCFA from the DFA was from 0 - 42 mm in males and 0 - 40 mm in females. The average distance of the LCFA from the DFA ranged from 0 - 45 mm. 

A CT evaluation in a 47-year-old male patient in France demonstrated that the MCFA emerged from the EIA, the LCFA came from the FA, and the DFA arose from the FA on the posteromedial side distally [29]. On the left side, the DFA originated from the medial side of the FA, whereas the LCFA came from the CFA at the same level.

Another case report regarding an 83-year-old Japanese male cadaver concluded that there was a common trunk of the deep circumflex iliac artery and part of the dLCFA at the transition from the EIA to the CFA under the inguinal ligament (IL) on the right side [30]. The MCFA and the remainder of the LCFA came from the DFA. As for the left limb, the MCFA, the inferior epigastric artery (IEA), and the obturator artery (OA) arose from the CFA, whereas the LCFA arose from the DFA. 

Marcucci et al. published a case report describing a 47-year-old female in Italy in which examination revealed a complete transposition of the femoral artery and vein, where the CFA was medial to the CFA [31]. On the left thigh of the female cadaver of Caucasian origin in Athens, two DFAs were found being at a distance of 5.4 cm and 9.1 cm of the MIP without circumflex arteries [32]. 

In a 53-year-old Indian male cadaver, the LCFA coming from the DFA, traversed deep to the posterior division of the FN, instead of the anterior [33]. Another branch from the FA, distal to the LCFA, ran parallel to the LCFA, imitating the usual course of the latter. Lastly, a case report on a cadaveric dissection in Turkey described the posterolateral origin of the MCFA from the FA [34]. 

Regarding the variations in the ALT flap's vascular anatomy, a total of 277 CTAs were retrospectively analyzed in India by Lim et al. who concluded that the dLCFA, the branch that most frequently vascularizes the ALT flap, arose from the LCFA in 87.1% of cases [35]. Less common sources were the DFA, the CFA, and the SFA. The same study displayed that the oblique branch, present in 47% of extremities, contributed to the blood supply of the ALT flap. This arose from the tLCFA in 46.1% of cases, from the dLCFA or from the LCFA itself. 

Another study conducted on 19 distally-based thigh flaps in 19 patients in China by Liu et al. illustrated that in eight out of the 19 cases, the source vessel of the ALT flap was the oblique branch [36]. In seven of these cases, it originated from the dLCFA and in one case from the tLCFA. A review of 110 ALT flaps in Taiwan by Lee et al. demonstrated that 69.1% of flaps were vascularized by the dLCFA, 9.1% from the tLCFA, and in 21.8% of the cases, the origin was dual [21]. 

A clinical study by Rozen et al. carried out on 44 patients who underwent ALT flap reconstruction showed that 16% of patients who underwent ALT flaps without preoperative CTA did not have any suitable perforators (> 1 mm) from which to harvest a flap supplied by the dLCFA [37]. In these cases, the ascending or transverse branch of the LCFA or MCFA were explored. Even if this was impossible, the contralateral leg was the next target. 

**Figure 2:**
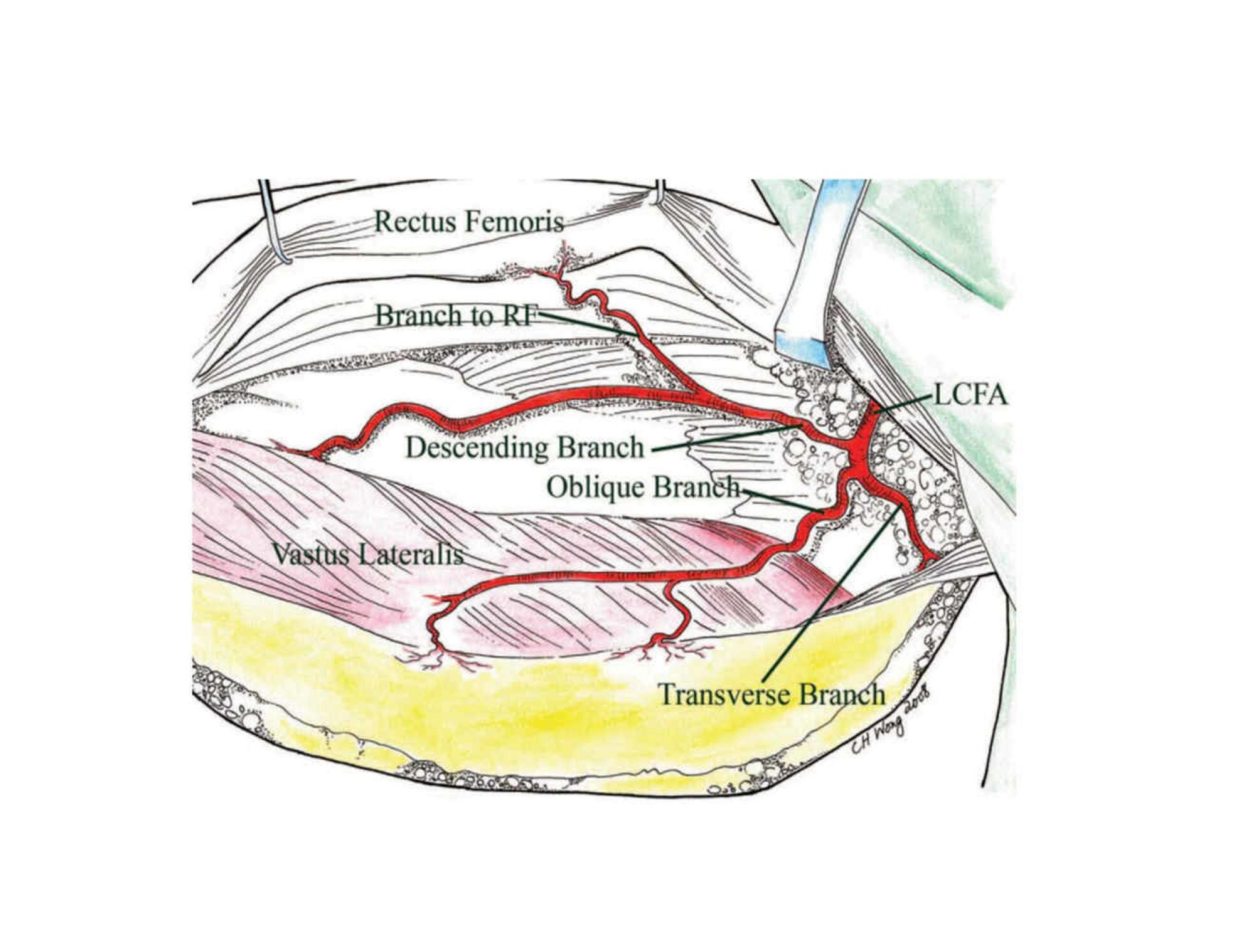
Myocutaneous flap based only on perforators from the oblique branch of the lateral circumflex femoral artery (LCFA) Reproduced from “Alternative vascular pedicle of the anterolateral thigh flap: the oblique branch of the lateral circumflex femoral artery” by Wong et al., Plast Reconstr Surg, 2009, 123:571-577 [38]. Reprinted with permission.

**Figure 3:**
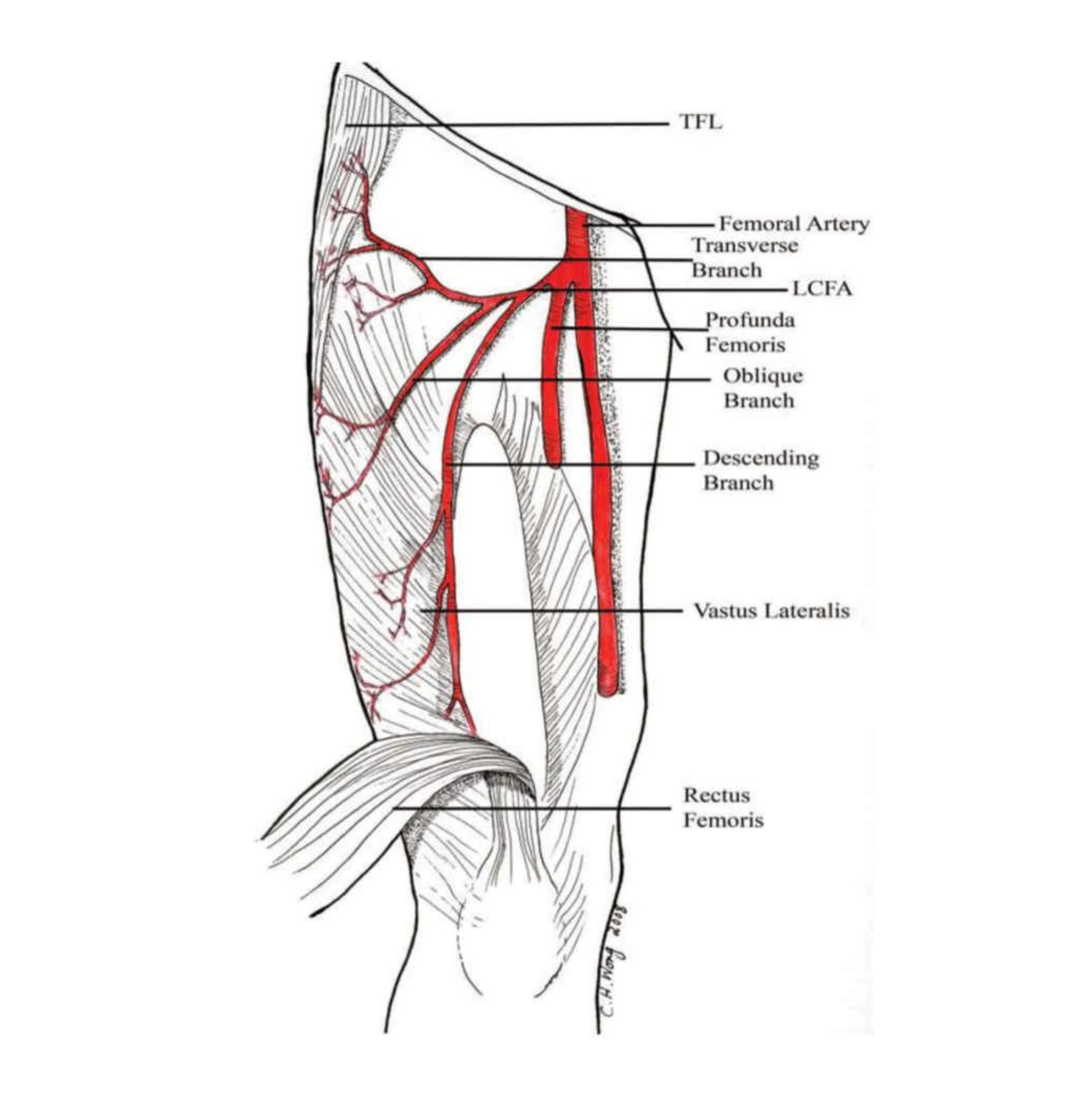
Oblique branch, originating from the lateral circumflex femoral artery (LCFA) Picture depicting the presence of an oblique branch, originating from the LCFA, that runs between the transverse and the descending branches of the LCFA and vascularizes the lateral thigh. TFL: tensor fascia latae. Reproduced from “Alternative vascular pedicle of the anterolateral thigh flap: the oblique branch of the lateral circumflex femoral artery” by Wong et al., Plast Reconstr Surg, 2009, 123:571-577 [38]. Reprinted with permission.

A prospective intraoperative study of the ALT flap in Singapore by Wong et al. concluded that in 31 cases out of 88, there was a distinct oblique branch, whereas in 12 cases, the flap was based on this branch (Figures 2, 3) [38]. In one out of 88 cases, no sizable perforator was found, obliging surgeons to harvest a flap in the vicinity of the incision, in the opposite thigh, or even a different flap. 

A cadaveric study in Thailand by Boonrod et al. revealed that the dLCFA derived in 80% of cases from the LCFA, 12% from the CFA, and 8% from the DFA [39]. It also demonstrated that the LCFA gave off a single dLCFA in 76% of cases, a double in 6%, a triple in 2%, whereas a common dLCFA with single split-end branches was apparent in 16% of the cases. 

Another study carried out in Taiwan by Lu et al. on 548 ALT flaps demonstrated that 55% of them required conversion to another flap due to small perforators (47% of the cases) or absent perforators (40% of the cases) [40]. 

A study by Seth et al., based on the CTA technique, depicted that the midpoint perforator was located in a surrounding distance of ± 2% of the midpoint of the total thigh-length only in 47% of cases [41]. The other perforators were at a distance of 52.7 mm proximal and 58.6 mm distal to the midpoint. It also displayed that the perforator vessel arose mostly from the LCFA. In 4% of cases, it derived from the SFA and in 1.1% from the CFA. 

Kekatpure et al. published a study on 25 patients who underwent free ALT flaps and concluded that, in 21 cases, perforators arose from the dLCFA and, in four cases, from the oblique branch of the LCFA [42]. 

An Italian study by Ribuffo et al., based on the preoperative CTA technique, showed that the perforator arose from the dLCFA in 90.69% of the cases and from the tLCFA in 9.31% of the cases [43]. 

A case report of a 45-year-old man in Taiwan described an ALT flap with two perforators from the LCFA without any connection with the dLCFA [44].

Our review underlines the fact that the point at which the DFA branches from the FA is highly variant. Our results revealed that the most common site of origin of the DFA was the posterolateral wall of the FA. The second most frequent type of origin of the DFA was the posterior wall of the FA in accordance with two Indian studies, whereas a third Indian study illustrated the lateral wall as the second one. A variance was also noticed in the pattern of origin of the LCFA and the MCFA. Except for one study, in which the MCFA of the right limbs originated most frequently from the CFA, the majority of the left MCFAs in the same study and the MCFAs in the other studies, came mostly from the PFA. Similarly, the LCFA originated principally from the DFA, with the CFA being the second most frequent source of origin. As for the predominant site of origin, it was the medial for the MCFA and the lateral for the LCFA. The dLCFA was the main source of the perforators of the ALT flap in the majority of the cases described. This arose most often from the LCFA, but the DFA, the CFA, and the SFA represent other probable sources. A further consequential result was that, in some cases, an oblique branch was recognizable, either contributing to the vascularization of the flap or constituting its sole pedicle. The tLCFA, the dLCFA, and, less regularly, the LCFA were recorded as potential origins of this oblique branch.

## Conclusions

The aim of our study was to collect and analyze all available information correlated to the anatomical characteristics of the DFA and its branches in order to ameliorate the current clinically pertinent knowledge. This information is vital for surgeons, interventional radiologists, and other medical professionals performing procedures in the femoral region. Furthermore, the use of ALT flaps has become an optimal option, especially for head and neck reconstruction. It is a multipurpose soft tissue flap that can be harvested as a fasciocutaneous or myocutaneous flap, whereas donor morbidity is strikingly minimal in spite of the availability of plenty of amounts of tissue. Notwithstanding that, the anatomical variations encountered may render this unreliable and risky. Preoperative CTA is an extremely reliable tool for displaying vascular anatomy and is superior to previous techniques, such as Doppler ultrasound. The use of CTA can contribute to the selection of the appropriate limb and perforator, thus enhancing flap design and operating outcomes. Excellent knowledge of anatomy can assist surgeons in surpassing uncertainties and safely harvest the flap. Several authors have demonstrated that in a significant number of patients, the skin of the ALT flap is supplied by a source other than the dLCFA, called the oblique branch. When present, this branch is of adequate size consisting of one artery and commonly two veins, thus permitting the acquisition of a viable and successful flap.

## References

[1] Sabnis AS (2013). Anatomical variations of profunda femoris artery. J Clin Res Letters.

[2] Vuksanovic-Bozaric A, Radojevic N, Muhovic D, Abramovic M, Radunovic M (2015). Significance of anatomical variations of the lateral circumflex femoral artery for the tensor fasciae latae flapping. Folia Morphol (Warsz).

[3] Vuksanović-Božarić A, Stefanović N, Pavlović S, Đurašković R, Ranđelović J (2007). Analysis of deep femoral artery origin variances on fetal material. Facta Univ Med Biol.

[4] Lippert H, Pabst R (2011). Arterial Variations in Man: Classification and Frequency. J.F. Bergmann-Verlag.

[5] Manjappa T, Prasanna LC (2014). Anatomical variations of the profunda femoris artery and its branches--a cadaveric study in South Indian population. Indian J Surg.

[6] Mamatha H, D’souza AS, Jessica S, Susani S (2012). A cadaveric study on the variations in the origin, course and branching pattern of the profunda femoris artery. Int J Cur Res Rev.

[7] Choy KW,  Kogilavani S,  Norshalizah M (2013). Tographical anatomy of the profunda femoris artery and the femoral nerve: normal and abnormal relationships. Clin Ter.

[8] Kanawati AJ (2014). Variations of the sciatic nerve anatomy and blood supply in the gluteal region: a review of the literature. ANZ J Surg.

[9] Lalović N, Cvijanović R, Malis M, Ilić M, Cuk M, Nikolić I (2013). Surgical anatomy of the initial segment of the lateral circumflex femoral artery (Article in Serbian). Med Pregl.

[10] Lin DT, Coppit GL, Burkey BB (2004). Use of the anterolateral thigh flap for reconstruction of the head and neck. Curr Opin Otolaryngol Head Neck Surg.

[11] Sinkeet SR, Ogeng’o JA, Elbusaidy H, Olabu BO, Irungu MW (2012). Variant origin of the lateral circumflex femoral artery in a black Kenyan population. Folia Morphol (Warsz).

[12] Üzel M, Tanyeli E, Yildirim M (2008). An anatomical study of the origins of the lateral circumflex femoral artery in the Turkish population. Folia Morphol (Warsz).

[13] Gautier E, Ganz K, Krügel N, Gill T, Ganz R (2000). Anatomy of the medial femoral circumflex artery and its surgical implications. J Bone Joint Surg Br.

[14] Güttler K, Pokorný D, Sosna A (2007). The role of understanding the media femoral circumflex artery course in total hip replacement (Article in Czech). Acta Chir Orthop Traumatol Cech.

[15] Langer R, Langer M, Scholz A, Astinet F, Schwetlick G, Felix R (1993). Femoral head perfusion in patients with femoral neck fracture and femoral head necrosis. J Belge Radiol.

[16] Filis K, Arhontovasilis F, Theodorou D (2007). Management of early and late detected vascular complications following femoral arterial puncture for cardiac catheterization. Hellenic J Cardiol.

[17] Locke MB, Zhong T, Mureau MA, Hofer SO (2012). Tug ‘O’ war: challenges of transverse upper gracilis (TUG) myocutaneous free flap breast reconstruction. J Plast Reconstr Aesthet Surg.

[18] Wei FC, Jain V, Celik N, Chen HC, Chuang DC, Lin CH (2002). Have we found an ideal soft-tissue flap? An experience with 672 anterolateral thigh flaps. Plast Reconstr Surg.

[19] Song YG, Chen GZ, Song YL (1984). The free thigh flap: a new free flap concept based on the septocutaneous artery. Br J Plast Surg.

[20] Kimata Y, Uchiyama K, Ebihara S, Nakatsuka T, Harii K (1998). Anatomic variations and technical problems of the anterolateral thigh flap: a report of 74 cases. Plast Reconstr Surg.

[21] Lee YC, Chen WC, Chou TM, Shieh SJ (2015). Anatomical variability of the anterolateral thigh flap perforators: vascular anatomy and its clinical implications. Plast Reconstr Surg.

[22] Koshima I, Fukuda H, Utunomiya R, Soeda S (1989). The anterolateral thigh flap; variations in its vascular pedicle. Br J Plast Surg.

[23] Lalović N, Mališ M, Korica M, Cvijanović R, Simatović M, Ilić M (2012). Origin of the medial circumflex femoral artery--a cadaver study. Med Glas (Zenica).

[24] Zlotorowicz M, Czubak-Wrzosek M, Wrzosek P, Czubak J (2018). The origin of the medial femoral circumflex artery, lateral femoral circumflex artery and obturator artery. Surg Radiol Anat.

[25] Vuksanović-Božarić A, Abramović M, Vučković L, Golubović M, Vukčević B, Radunović M (2018). Clinical significance of understanding lateral and medial circumflex femoral artery origin variability. Anat Sci Int.

[26] Rajani SJ, Ravat MK, Rajani JK, Bhedi AN (2015). Cadaveric study of profunda femoris artery with some unique variations. J Clin Diagn Res.

[27] Prakash Prakash, Kumari J, Kumar Bhardwaj A, Jose BA, Kumar Yadav S, Singh G (2010). Variations in the origins of the profunda femoris, medial and lateral femoral circumflex arteries: a cadaver study in the Indian population. Rom J Morphol Embryol.

[28] Nasr A, Badawoud M, Al-Hayani A, Hussein A (2014). Origin of profunda femoris artery and its circumflex femoral branches: anatomical variations and clinical significance. Folia Morphol (Warsz).

[29] Rusu M, Ilie A, Brezean I (2017). Human anatomic variations: common, external iliac, origin of the obturator, inferior epigastric and medial circumflex femoral arteries, and deep femoral artery course on the medial side of the femoral vessels. Surg Radiol Anat.

[30] Nasu H, Chiba S (2009). Rare case of femoral artery ramification and origin of the obturator artery. Anat Sci Int.

[31] Marcucci G, Antonelli R, Accrocca F, Siani A (2010). A rare anomaly of the femoral vessels: complete transposition of the femoral artery and vein. Interact Cardiovasc Thorac Surg.

[32] Tsoucalas G, Panagouli E, Fiska A, Troupis T, Venieratos D (2018). A rare double profunda femoris artery in a female cadaver. Anat Cell Biol.

[33] Goel S, Arora J, Mehta V, Sharma M, Suri R, Rath G (2015). Unusual disposition of lateral circumflex femoral artery: anatomical description and clinical implications. World J Clin Cases.

[34] Ciftcioğlu E, Kale A, Kopuz C, Edizer M, Aydin E, Demir MT (2009). Medial circumflex femoral artery with different origin and course: a case report and review of the literature. Folia Morphol (Warsz).

[35] Lim S, Atwi N, Long S, Toshav A, Lau F (2018). Variations in the anterolateral thigh flap's vascular anatomy in African Americans. J Reconstr Microsurg.

[36] Liu Y, Ding Q, Zang M, Yu S, Zhu S, Chen B, Zhang J (2017). Classification and application of the distally-based thigh flap based on the lateral circumflex femoral artery system. Ann Plast Surg.

[37] Rozen W, Ashton M, Pan W (2009). Anatomical variations in the harvest of anterolateral thigh flap perforators: a cadaveric and clinical study. Microsurgery.

[38] Wong C, Wei F, Fu B, Chen Y, Lin J (2009). Alternative vascular pedicle of the anterolateral thigh flap: the oblique branch of the lateral circumflex femoral artery. Plast Reconstr Surg.

[39] Boonrod A, Thammaroj T, Jianmongkol S, Prajaney P (2016). Distal anastomosis patterns of the descending branch of the lateral circumflex femoral artery. J Plast Surg Hand Surg.

[40] Lu JC, Zelken J, Hsu C, Chang N, Lin C, Wei F, Lin C (2015). Algorithmic approach to anterolateral thigh flaps lacking suitable perforators in lower extremity reconstruction. Plast Reconstr Surg.

[41] Seth R, Manz R, Dahan I, Nuara M, Meltzer N, McLennan G, Alam D (2011). Comprehensive analysis of the anterolateral thigh flap vascular anatomy. Arch Facial Plast Surg.

[42] Kekatpure V, Trivedi N, Shetkar G, Manjula BV, Mohan A, Kuriakose M (2011). Single perforator based anterolateral thigh flap for reconstruction of large composite defects of oral cavity. Oral Oncol.

[43] Ribuffo D, Atzeni M, Saba L, Milia A, Guerra M, Mallarini G (2009). Angio computed tomography preoperative evaluation for anterolateral thigh flap harvesting. Ann Plast Surg.

[44] Wong CH, Wei FC (2010). Anterolateral thigh flap. Head Neck.

[45] Tomaszewski KA, Henry BM, Vikse J (2017). Variations in the origin of the deep femoral artery: a meta-analysis. Clin Anat.

